# Inhibition of the Monocarboxylate Transporter 1 (MCT1) Promotes 3T3-L1 Adipocyte Proliferation and Enhances Insulin Sensitivity

**DOI:** 10.3390/ijms23031901

**Published:** 2022-02-08

**Authors:** Tracey Bailey, Ainhoa Nieto, Patricia McDonald

**Affiliations:** 1 Department of Cancer Physiology, Moffitt Cancer Center, 12902 Magnolia Drive, Tampa, FL 33612, USA; Tracey.Bailey@moffitt.org; 2 Primary Pharmacology Group, Discovery Sciences, Pfizer, Inc., 445 Eastern Point Rd, Groton, CT 06340, USA; Ainhoa.Nieto@pfizer.com

**Keywords:** adipocytes, hyperplasia, insulin sensitivity, MCT1, cell cycle, proliferation

## Abstract

Enlarged, hypertrophic adipocytes are less responsive to insulin and are a hallmark feature of obesity, contributing to many of the negative metabolic consequences of excess adipose tissue. Although the mechanisms remain unclear, the adipocyte size appears to be inversely correlated with insulin sensitivity and glucose tolerance, wherein smaller adipocytes are insulin-sensitive and larger adipocytes develop insulin resistance and exhibit an impaired glucose uptake. Thus, pharmacological strategies aimed at regulating adipocyte hypertrophy (increase in adipocyte size) in favor of promoting hyperplasia (increase in adipocyte number) have the potential to improve adipocyte insulin sensitivity and provide therapeutic benefits in the context of metabolic disorders. As white adipose tissue can metabolize large amounts of glucose to lactate, using transcriptomics and in vitro characterization we explore the functional consequences of inhibiting monocarboxylate transporter 1 (MCT1) activity in fully differentiated adipocytes. Our studies show that the pharmacological inhibition of MCT1, a key regulator of the cellular metabolism and proliferation, promotes the re-entry of mature adipocytes into the cell cycle. Furthermore, we demonstrate that inhibitor-treated adipocytes exhibit an enhanced insulin-stimulated glucose uptake as compared with untreated adipocytes, and that this outcome is dependent on the cyclin-dependent kinase 1 (CDK1) activity. In summary, we identify a mechanism though which MCT1 inhibition improves the insulin sensitivity of mature adipocytes by inducing cell cycle re-entry. These results provide the foundation for future studies investigating the role MCT1 plays in adipocyte hyperplasia, and its therapeutic potential as a drug target for obesity and metabolic disease.

## 1. Introduction

Over the last four decades, there has been a dramatic increase in the prevalence of obesity, with recent reports (2017–2018) [1] indicating that >73% of the adult US population is overweight, with a body mass index (BMI) of >25 kg/m^2^, and 42.5% designated as obese (BMI > 30 kg/m^2^). Concomitant with the trends in obesity is the marked increase in the prevalence of type 2 diabetes mellitus (T2DM), where more than 85% of T2DM patients are also obese [2]. T2DM is a complex metabolic disorder characterized by hyperglycemia arising from the combination of an impaired insulin secretion, increased hepatic glucose production, and a decreased insulin-mediated glucose uptake (insulin resistance) [3]. In addition to the epidemiological evidence establishing obesity as the leading risk factor for T2DM [4], the molecular mechanisms though which obesity contributes to the development of insulin resistance in insulin-responsive tissues (i.e., skeletal muscle, adipose tissue, and the liver) can be largely credited to the inability of mature white adipocytes to perpetually expand to accommodate excess energy in the form of triglycerides. To compensate, ‘lipid overflow’ into ectopic sites (i.e., liver, kidney, skeletal muscle, and pancreas) occurs at the expense of inducing lipotoxicity and insulin resistance [5].

After reaching lipid saturation, adipocytes can undergo hypertrophy (increase in adipocyte size) or hyperplasia (increase in number of adipocytes) to accommodate for increased triglyceride storage needs [6]. Although a subset of obese individuals referred to as metabolically ‘healthy’ obese maintain adipose tissue expandability though hyperplasia, adipose tissue expansion in the majority of obese individuals occurs primarily though hypertrophy [7]. In this context, enlarged adipocytes have a profound impact on the metabolic health of adipose tissue and in regulating insulin sensitivity [8], glucose uptake, and inflammation [9]. It has long been appreciated that an increased adipocyte size correlates to systemic insulin resistance [10], while smaller adipocytes retain insulin sensitivity. As adipocyte hypertrophy contributes to many of the adverse metabolic events associated with obesity, inducing hyperplasia or cell division in hypertrophic adipocytes has the potential to redistribute the lipid content and improve the overall metabolic health of obese individuals. Hence, it has recently been suggested that inducing hyperplasia to improve the metabolic ‘health’ of adipocytes may be an alternative therapeutic strategy to weight loss to treat insulin resistance and related metabolic diseases [10].

Under normal physiological conditions, adipocyte hyperplasia occurs in response to the secretion of paracrine growth factors, leading to an increase in the number of preadipocytes within the adipose tissue depot and their subsequent differentiation into mature adipocytes. This process is dependent on the tight regulation of several cell cycle-related events, including the growth arrest of proliferating preadipocytes, the coordinated re-entry into the cell cycle though mitotic clonal expansion, and, finally, terminal differentiation [10]. However, it has been proposed that mature adipocytes also contribute to hyperplasia either though de-differentiation [11], subsequent proliferation and re-differentiation [10], or re-entry of mature adipocytes into the cell cycle [12,13,14]. Thus, proteins or signaling pathways that regulate the adipocyte growth arrest and cell cycle could promote hyperplasia and have the potential to serve as therapeutic targets for the treatment of various metabolic disorders.

For this, we propose one such protein, the monocarboxylate transporter 1 (MCT1), one of 14 members of the *SLC16a* solute carrier gene family encoding monocarboxylate transporters (MCTs). Within this subfamily, MCT1, MCT2, MCT3, and MCT4 have been identified as proton-linked, bidirectional transporters responsible for the influx and efflux of monocarboxylates such as lactate, pyruvate, ketone bodies, and certain drugs across the plasma membrane [15]. In addition to regulating lactate trafficking, MCT1 is a well-established target of c-Myc, an oncogenic transcription factor that drives continuous cell growth and division [16], and is highly upregulated in proliferating cells [17]. Interestingly, MCT1 expression has also been reported to increase over the course of adipocyte differentiation owing to an increased lactate flux as preadipocytes mature into adipocytes [18]. Adipocytes are highly glycolytic even under conditions of excess oxygenation and contribute significantly to circulating lactate levels, particularly in obese individuals [19]. Furthermore, serum lactate levels have been shown to directly correlate with insulin resistance in obese and non-obese individuals [19]. While an enhanced lactate metabolism and MCT1 expression are associated with adipocyte differentiation, proliferation, and systemic insulin resistance, the relationship between these processes and the influence of modulating an MCT1-mediated lactate flux in adipocytes is unknown.

By regulating the intracellular lactate concentration, MCT1 also plays an important role in regulating redox homeostasis [20,21]. In adipocytes, lactate abundance and MCT1 function have been shown to impact redox signaling mechanisms with widespread biological consequences. This is primarily accredited to the function of lactate as an electron donor, enabling the reduction in NAD^+^ to NADH though its lactate dehydrogenase catalyzed oxidation to pyruvate [22]. While it is known that redox signaling is critical to cell cycle progression and growth arrest [23], it has recently been reported that MCT1-mediated lactate transport induces plasticity and mitochondrial biogenesis as a mechanism to alleviate redox pressure in mature adipocytes [24].

In this study, using the classical white adipocyte cell model, murine 3T3-L1 cells, we demonstrate that treatment with a small molecule inhibitor of MCT1 (AZD3965) results in the re-entry of mature adipocytes into the cell cycle. We report that the adipogenic profile of differentiated 3T3-L1 cells treated with an MCT1 inhibitor is altered at the mRNA, protein, and phenotypic levels, wherein these cells exhibit distinct transcript and protein expression signatures, as well as a decrease in the lipid content and enhanced insulin sensitivity. Thus, inhibiting MCT1 activity in mature adipocytes may serve as a novel mechanism to induce re-entry of fully differentiated adipocytes into the cell cycle with the potential to promote adipocyte proliferation and hyperplasia. Our results highlight the need to further investigate the role MCT1 plays in adipocyte metabolism and its potential as a therapeutic drug target for insulin resistance in metabolic disorders.

## 2. Results

### 2.1. Inhibition of MCT1 in 3T3-L1 Cells Hinders Adipocyte Lipid Accumulation

**Figure 1:**
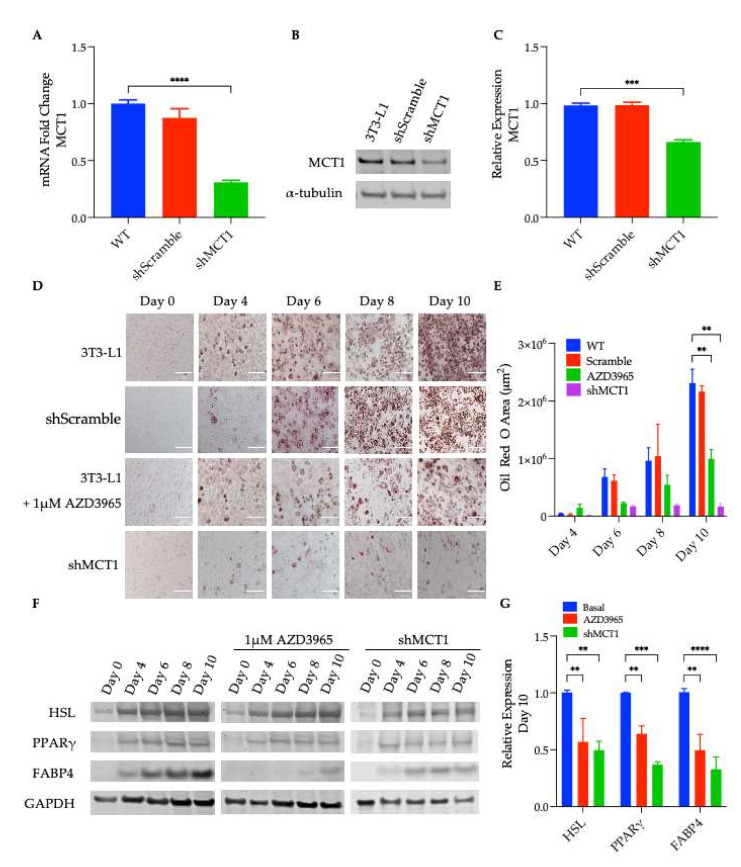
Influence of monocarboxylate transporter 1(MCT1) inhibition on preadipocyte differentiation. (**A**) cDNA generated from 3T3-L1 wildtype (WT), 3T3-L1-shMCT1, and 3T3-L1-shScramble was subjected to RT-qPCR with MCT1 primers (**** *p* < 0.0001). (**B**) Cell lysates were subjected to Western blot analysis using anti-MCT1 antibodies (*** *p* < 0.001). (**C**) Quantification of MCT1 protein expression. (**D**) Oil Red O staining of 3T3-L1 (WT) cells treated with or without 1 µM AZD3965, 3T3-L1-shMCT1, and 3T3-L1-shScramble during differentiation. Scale bars depict 100 µm. (**E**) Quantification of Oil Red O staining. (**F**) Immunoblotting of hormone sensitive lipase (HSL), peroxisome proliferator-activated receptor γ (PPARγ), and fatty acid-binding protein 4 (FABP4) in 3T3-L1 (WT) and 3T3-L1-shMCT1 cells undergoing differentiation in the absence or presence of 1 µM AZD3965 at indicated time points. (**G**) Quantification of HSL, PPARγ, and FABP4 expression on day 10. (** *p* < 0.01). Values presented represent mean ± SEM for 3 biological replicates.

Murine embryonic fibroblast cells, 3T3-L1, were cultured and differentiated into mature lipid-laden adipocytes according to well-established protocols [25]. In agreement with previous reports, MCT1 expression in 3T3-L1 cells was confirmed at both the mRNA and protein levels (Figure 1A–C, respectively). To enable the study of genetically inhibiting MCT1 expression, 3T3-L1 cells transduced with short hairpin RNA directed against MCT1 (shMCT1) or a scrambled RNA (shScramble) were used to generate stable 3T3-L1 cell lines depleted of MCT1, and an shNA 3T3-L1 control cell line (3T3-L1-shMCT1 and 3T3-L1-shScramble, respectively). The successful knockdown of MCT1 expression at both the mRNA and protein levels in 3T3-L1-shMCT1 was demonstrated with a reverse transcription–quantitative polymerase chain reaction (RT-qPCR) and immunoblotting (Figure 1A–C, respectively).

To determine the effect of pharmacological inhibition of MCT1 on adipocyte differentiation, 3T3-L1 cells were differentiated with or without 1 µM AZD3965, the previously described potent and selective small-molecule MCT1 inhibitor [26]. As expected, by day six of the differentiation process, both control 3T3-L1 and shScramble cells began forming lipid droplets as measured by Oil Red O staining (Figure 1D,E). Conversely, 3T3-L1 cells that were differentiated in the presence of 1 µM AZD3965 and 3T3-L1-shMCT1 cells contained significantly less lipid over the course of differentiation (Figure 1D; third and fourth panels, respectively). The quantification of the Oil Red O area (i.e., lipid content) is presented in Figure 1E.

### 2.2. MCT1 Inhibition Suppresses Induction of the Adipogenic Gene Expression in Preadipocytes

Shortly after exposure to differentiation media (containing insulin, dexamethasone, and 3-isobutyl-1-methylxanthine (IBMX); see ‘Differentiation Protocol’ in the Materials and Methods Section), the adipogenic gene expression program was induced, upregulating various transcription factors and adipose-specific genes. Thus, the protein expression of the transcription factor peroxisome proliferator-activated receptor γ (PPARγ), considered to be the master regulator of adipogenesis, and the PPARγ-regulated adipocyte markers, fatty acid-binding protein 4 (FABP4/aP2) [27] and hormone-sensitive lipase (HSL) [28], were analyzed. As expected, the expression of these adipocyte-related proteins increased as the differentiation progressed in untreated 3T3-L1 cells and in 3T3-L1-shscramble cells. However, in 3T3-L1 cells differentiated in the presence of AZD3965 and shMCT1 containing 3T3-L1 cells, the expression levels of PPARγ, HSL, and FABP4 were significantly attenuated (Figure 1F,G).

### 2.3. Mature Adipocytes Show Reduced Lipid Content, but Remain Differentiated after MCT1 Inhibition

**Figure 2:**
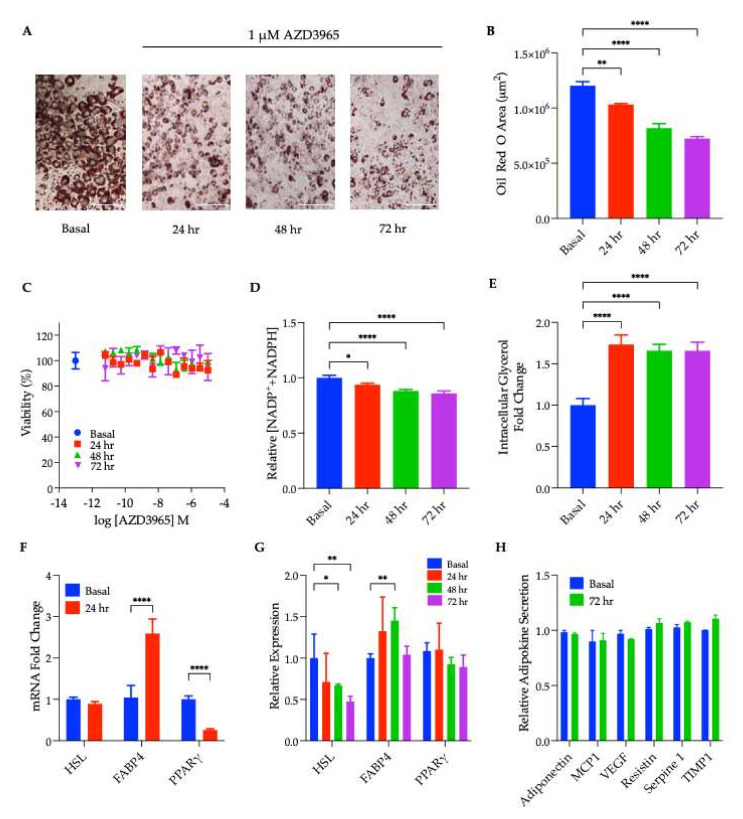
MCT1 inhibition in fully differentiated adipocytes. (**A**) Oil Red O staining of differentiated 3T3-L1 (WT) cells treated with or without 1 µM AZD3965 for 24 h, 48 h, or 72 h. (**B**) Quantification of Oil Red O staining (**** *p* < 0.0001) (*** *p* < 0.001) (** *p* < 0.01). (**C**) Cell viability of differentiated 3T3-L1 (WT) cells treated with or without 1 µM AZD3965 for 24 h, 48 h, or 72 h. (**D**) Relative intracellular concentrations of NADP+ and NADPH following treatment with or without 1 µM AZD3965 for 24 h, 48 h, or 72 h (* *p* < 0.05). (**E**) Intracellular glycerol was quantified following treatment with or without 1 µM AZD3965 for 24 h, 48 h, or 72 h. (**F**) mRNA levels of HSL, PPARγ, and FABP4 in 3T3-L1 cells treated with or without 1 µM AZD3965 for 24 h (** *p* < 0.01). (**G**) Quantifications of immunoblotting experiments (immunoblots showing relative protein expression levels of HSL, PPARγ, and FABP4 in differentiated 3T3-L1 (WT) cells treated with 1 µM AZD3965 for 0 to 72 h (immunoblots can be found in Supplementary Figure S1A. (**H**) Differentiated adipocytes treated with or without 1 µM AZD3965 for 72 h. Fresh media was conditioned for 24 h prior to collection and analysis with an adipokine antibody array (antibody array blots can be found in Supplementary Figure S1B). Quantification of the resulting chemiluminescent signals are presented in (H). Values presented represent mean ± SEM for 3 biological replicates.

Similar experiments to those carried out in differentiating preadipocytes were performed to assess the impact of MCT1 inhibition on fully differentiated, ‘mature’ adipocytes. Differentiated adipocytes showed a progressive decrease in the lipid content following a 1 µM AZD3965 treatment (Figure 2A,B; microscopy and quantification, respectively). A viability assay performed on these cells demonstrated that the reduction in the lipid content was not due to AZD3965-induced cytotoxicity (Figure 2C). After cell death was eliminated as a potential explanation for the observed depletion in the adipocyte lipid content, changes in the lipid metabolism were examined. As nicotinamide adenine dinucleotide phosphates (NADP^+^/NADPH) are critical cofactors for lipid biosynthesis, changes in the bioavailability of NADP^+^ and NADPH were monitored. A subtle yet significant decrease in the concentration of this cofactor species was observed following 24 h, 48 h, and 72 h of MCT1 inhibition (Figure 1E), suggesting that AZD3965-treated cells were unable to support the high rates of lipogenesis characteristic of adipocytes. The influence of MCT1 inhibition on rates of lipolysis, the primary mechanism of triglyceride catabolism into glycerol and free fatty acids in adipocytes, was also probed. The AZD3965 treatment significantly increased intracellular glycerol levels at all time points measured (Figure 2E), indicating that MCT1 inhibition resulted in a significant increase in cellular lipolysis.

In addition to assessing changes in the lipid content and metabolism in MCT1 inhibitor-treated cells, transcript and protein expression levels of the aforementioned adipogenic proteins, PPARγ, FABP4, and HSL, were also assessed following the inhibitor treatment (Figure 2F,G and Supplementary Figure S1A) to determine if these adipocytes remained differentiated following the AZD3965 treatment. Although the RT-qPCR demonstrated a significant decrease in PPARγ transcript levels in mature adipocytes following 24 h of treatment with AZD3965 (Figure 2F), no changes in PPARγ protein expression were observed following up to 72 h of AZD3965 treatment (Figure 2G). On the other hand, changes in transcript levels of FABP4 did correlate with corresponding changes in protein expression. Specifically, FABP4 mRNA levels were significantly increased following 24 h of MCT1 inhibition (Figure 2F), and this was reflected at the protein level for up to 48 h of compound treatment; however, by 72 h, this increase was attenuated (Figure 2G). Conversely, MCT1 inhibition resulted in a significant decrease in HSL expression following 24–72 h of compound treatment (Figure 2G). In addition to monitoring changes in the expression of these adipogenic proteins, changes in adipokine secretion following treatment with AZD3965 were also assessed utilizing an adipokine antibody microarray. Conditioned media collected from adipocytes that had been treated with or without AZD3965 for 72 h showed no significant differences in adipokine levels (Figure 2H and Supplementary Figure S1B), supporting the premise that adipocytes remain differentiated following MCT1 inhibition.

### 2.4. RNA Sequencing Reveals Transcriptional Consequences of MCT1 Inhibition in Adipocytes

**Figure 3:**
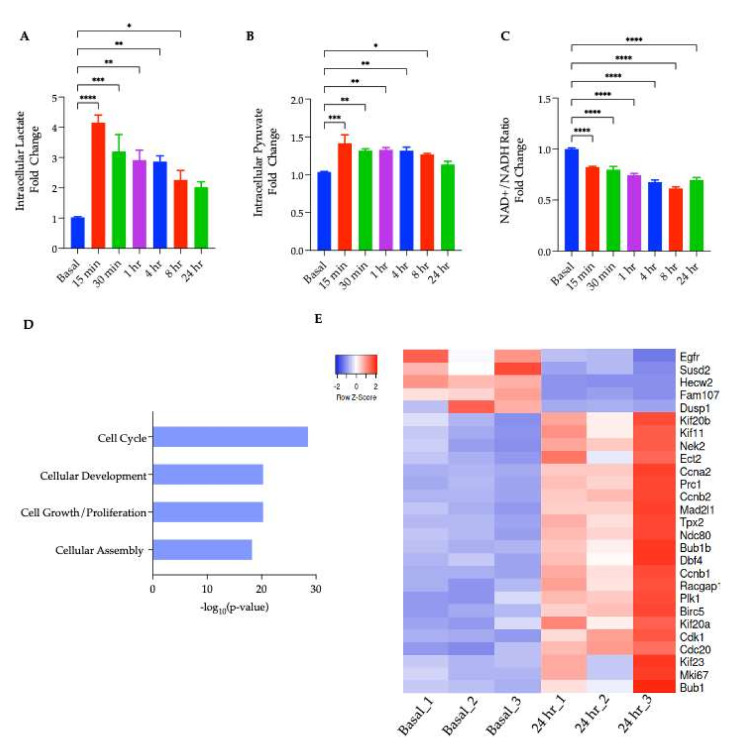
Phenotypic and transcriptomic analyses of adipocytes following MCT1 inhibition. (**A**) Intracellular lactate levels of differentiated adipocytes treated with 1 µM AZD3965 for 15 min to 24 h. (**** *p* < 0.0001) (*** *p* < 0.001) (** *p* < 0.01) (* *p* < 0.05) (**B**) Intracellular pyruvate levels of differentiated adipocytes treated with 1 µM AZD3965 for 15 min to 24 hr. (**C**) Following the same treatment paradigm, intracellular NAD^+^ and NADH were quantified and are presented as a ratio. (**D**) Significantly altered molecular functions predicted by IPA plotted against significance. (**E**) Heat map of transcript level changes in DEGs involved in cell cycle. Values presented represent mean ± SEM for 3 biological replicates.

Because the major role of MCT1 is the transport of lactate, pyruvate, and other monocarboxylates across the plasma membrane, the intracellular lactate concentration was measured at discrete time points ranging from 15 min to 24 h of the 1 µM AZD3965 treatment. Within 15 min of treatment, a significant increase in the intracellular lactate was observed (Figure 3A) and was maintained though the first 4 h of the AZD3965 treatment. With the export inhibited, lactate could be disposed of though its oxidation back into pyruvate or its direct, complete oxidation in the mitochondria. To examine these potential fates of lactate, changes in intracellular pyruvate and the intracellular NAD^+^/NADH ratio, often used as a proxy for cellular pyruvate/lactate [22], were monitored over the same time course. It was observed that MCT1 inhibition resulted in a significant increase in intracellular pyruvate following the first 8 h of treatment (Figure 2B), and a significant change in the ratio of NAD^+^/NADH across all time points measured (Figure 3C). Specifically, a significant decrease in the NAD^+^/NADH ratio was observed for the first 8 h of AZD3965 treatment indicating an increase in lactate relative to pyruvate. These results suggest that the AZD3965 treatment led to a significant increase in both intracellular lactate and pyruvate and a relative increase in lactate compared to pyruvate.

After 24 h of AZD3965 treatment, intracellular lactate levels were diminished to values approximately two-fold above basal, and intracellular pyruvate levels were not significantly altered (Figure 3A,B), suggesting that differentiated 3T3-L1 cells adapted within this time period to manage high intracellular lactate levels and a change in the NAD^+^/NADH ratio resulting from MCT1 inhibition. These findings, along with the previously observed significant decrease in the intracellular lipid content, were suggestive of transcriptional changes taking place within 24 h. To interrogate the consequences of MCT1 inhibition in mature adipocytes, we conducted a transcriptomics analysis using RNA sequencing (RNA-seq), followed by a comprehensive pathway analysis of adipocytes treated with or without 1 µM AZD3965 for 24 h.

Results from the RNA-seq experiment indicated that 277 genes were significantly (*p* < 0.05) differentially expressed following MCT1 inhibition. The pathway analysis conducted using the Ingenuity Pathway Analysis (IPA) revealed that these differentially expressed genes, including the cyclin-dependent kinase 1 (CDK1) [29], polo-like kinase (PLK1) [30], and DNA topoisomerase 2-α (TO2A) [31], among others, were largely implicated in the regulation of cell cycle and mitosis (Figure 3D). Genes involved in the progression of the cell cycle showed a significant upregulation in treated adipocytes, while genes that inhibited the cell cycle progression (e.g., DUSP1) [32] were significantly decreased (Figure 3E). Furthermore, molecular functions predicted to be significantly altered included cellular development, cell growth, and proliferation, as well as cellular assembly and organization (Figure 3D). The pathway analysis also predicted the activation of cytokinesis (the cytoplasmic division of a cell at the end of mitosis or meiosis, bringing about the separation into two daughter cells [33]). Taken together, a transcriptional fingerprint characterized by the upregulation of mRNAs related to the cell cycle and proliferation suggested potential for adipocytes to re-enter the cell cycle.

### 2.5. Adipocytes Re-Enter Cell Cycle following MCT1 Inhibition

**Figure 4:**
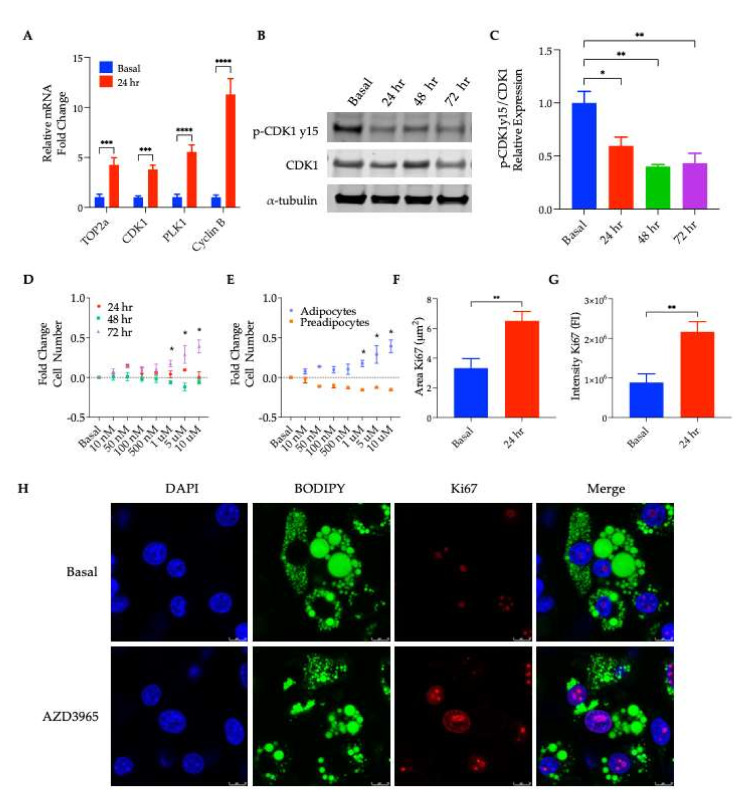
Analysis of adipocyte proliferation. (**A**) RT-qPCR analysis of mRNA levels of indicated genes performed on cDNA of adipocytes differentiated with or without1 µM AZD3965 for 24 h (**** *p* < 0.001), (*** *p* < 0.001). (**B**) p-CDK1y15, total CDK1, and α-tubulin expression in cell lysates collected from differentiated 3T3-L1 treated with or without 1 µM AZD3965 for 24 h, 48 h, or 72 h. (**C**) quantification of relative p-Tyr15-CDK1 expression normalized to total CDK1 and α-tubulin (** *p* < 0.01), (* *p* < 0.05). (**D**) Proliferation of differentiated 3T3-L1 cells treated with indicated concentrations of AZD3965 for 24 h, 48 h, or 72 h. (**E**) Assessment of proliferation in preadipocytes, differentiated 3T3-L1, and 3T3-L1-shMCT1 cells treated with the indicated concentrations of AZD3965 for 72 h. Quantification of (**H**) where (**F**) average pixel area of Ki67 (Cy5) per nuclei was calculated and presented in microns and (**G**) fluorescence intensity of Ki67 (Cy5) per nuclei was calculated. Values presented represent mean ± SEM for 3 biological replicates. (**H**) Differentiated adipocytes treated with or without 1 µM AZD3965 for 24 h were fixed and stained, and nucleus, Ki67, and lipid droplets visualized using confocal microscopy. DAPI nucleus/DNA staining (blue); BODIPY lipid droplet staining (green); Ki67 staining (red). Scale bars depict 10 µm.

To validate the results from RNA-seq, an RT-qPCR was performed, and it was confirmed that the MCT1 inhibitor treatment induced a significant upregulation of genes known to promote cell cycle progression, including CDK1, PLK1, TOP2A, and Cyclin B (Figure 4A). Of these genes, CDK1 activity is known to be largely regulated by post-translational modifications with differential influences on the progression of the cell cycle depending on certain phosphorylation and dephosphorylation events [34]. Therefore, CDK1 was probed for changes in protein expression and phosphorylation. While there was no change in the total CDK1 protein expression following treatment with an MCT1 inhibitor for up to 72 h, a significant decrease in CDK1 phosphorylation at Tyr15 was observed (Figure 4B,C). The phosphorylation of Tyr15 maintained CDK1 in an inactive state [35]. Thus, the decrease in Tyr15 phosphorylation following the AZD3965 treatment indicated an increase in the active form of CDK1, known to promote mitosis [36], supporting the hypothesis that MCT1 inhibition may promote adipocyte cell cycle progression and proliferation (i.e., hyperplasia).

To assess changes in adipocyte proliferation, a fluorescent DNA dye-based assay was used to determine if a change in the cell number occurred following MCT1 inhibition. After a 72 h treatment, a significant increase in the total cell number was observed for all concentrations of AZD3965 treatments of ≥1 µM (Figure 4D). To assess if the observed phenotype was specific to differentiated adipocytes or could be attributed to preadipocytes present in the cell culture even after rigorous differentiation, proliferation assays were also performed on preadipocytes. As shown in Figure 4E, the compound treatment had no effect on proliferation in preadipocytes, confirming that the proliferation phenomenon is unique to mature adipocytes.

Further studies using confocal microscopy were conducted to capture the proliferation of MCT1 inhibitor-treated mature adipocytes. Following 24 h of a 1 µM AZD3965 treatment, a significant increase in the expression of Ki67 (a widely-used marker of proliferation [37]) was observed when compared to untreated adipocytes. As seen in Figure 4H, the Ki67 expression was enhanced in the AZD3965-treated adipocytes as visualized by co-staining with BODIPY (a lipophilic green fluorescent dye used to stain lipid droplets, convenient for double fluorescence labeling in adipocytes), supporting the hypothesis that MCT1 inhibition promotes re-entry into the cell cycle and the subsequent proliferation of mature adipocytes. The quantification of Ki67 expression was performed by analyzing 1000 nuclei per experimental condition captured by 12 randomized images for the area and intensity of Ki67 (i.e., signal in the Cy5 channel) normalized by the nuclei number per image (Figure 4F,G).

### 2.6. MCT1 Inhibition Enhances Adipocyte Glucose Uptake in a CDK1-Dependent Manner

**Figure 5:**
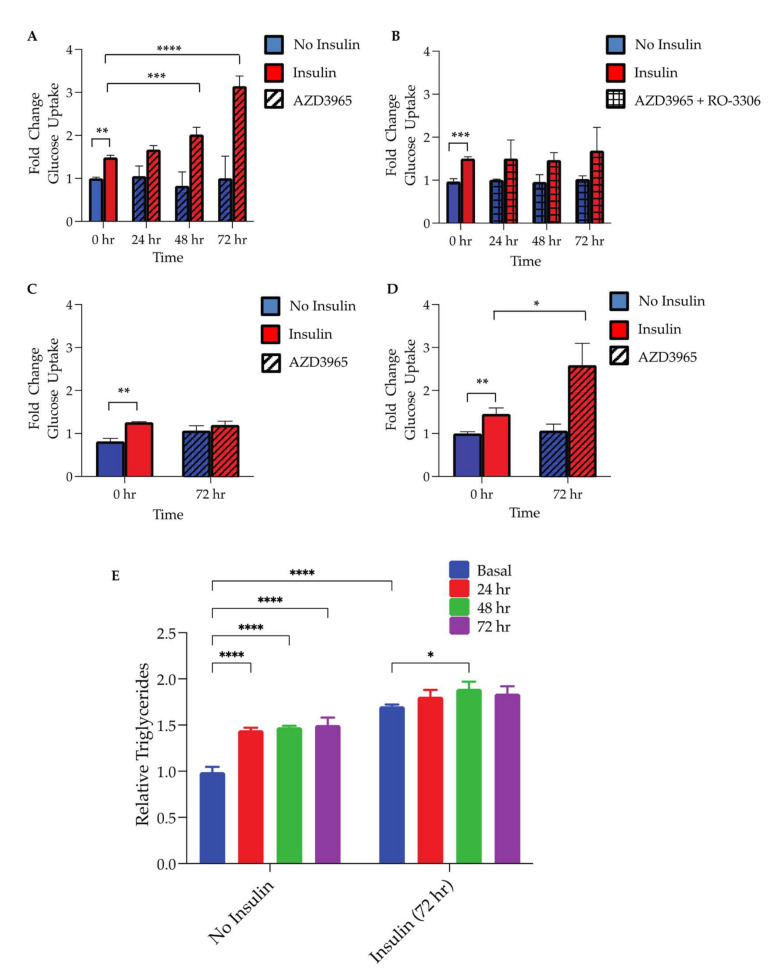
Insulin responsiveness. Glucose uptake in differentiated 3T3-L1 (WT) cells. (**A**) Glucose uptake in 3T3-L1 cells treated with 1 µM AZD3965 for 0 h (solid bars) or 24 h, 48 h, and 72 h (striped bars) were treated with (red bars) or without (blue bars) insulin for 30 min (**** *p* < 0.001), (*** *p* < 0.001), (** *p* < 0.01). (**B**) Experiment performed in (**A**) repeated in the presence of 10 µM RO-3306 (checked bars). (**C**) Experiment performed as in (**A**) with 3T3-L1 shMCT1 cells. (**D**) Experiment performed as in (**A**) with 3T3-L1-shScramble cells (** *p* < 0.01), (* *p* < 0.05). (**E**) Differentiated adipocytes were treated with or without (basal) 1 µM AZD3965 for 24 h, 48 h, or 72 h, as indicated. Adipocytes were then treated with or with insulin for 72 h. Following the treatment time course, total triglyceride content was assessed and is presented as a fold change normalized to basal (without AZD3965 and no insulin). Values presented represent mean ± SEM for 3 biological replicates.

A major metabolic benefit of inducing adipocyte hyperplasia is an increase in the insulin-stimulated glucose uptake. In order to evaluate the potential impact of MCT1 inhibition in mature adipocyte insulin sensitivity, alterations in glucose uptake rates were monitored. While the 1 µM AZD3965 treatment for up to 72 h had no significant impact on the non-insulin-stimulated glucose uptake (Figure 5A; blue bars), there was a significant increase in the insulin-stimulated glucose uptake following 48 h and 72 h of the MCT1 inhibitor treatment (Figure 5A; red bars). Trends toward an increase in the glucose uptake were also observed following 24 h of the AZD3965 treatment; however, this increase did not reach statistical significance. To determine if this effect was related to or distinct from the observed phenotype of cell cycle re-entry, the specific CDK1 inhibitor RO-3306 [38] was utilized. RO-3306 is known to induce growth arrest though CDK1 inhibition by preventing cells from entering mitosis. Differentiated adipocytes co-treated with RO-3306 (10 µM) and AZD3965 (1 µM) remained insulin responsive; however, the effects of AZD3965 were attenuated (Figure 5B).

To investigate if insulin handling was modified in adipocytes with decreased MCT1 expression, 3T3-L1-shMCT1 cells were differentiated and subjected to similar glucose uptake experiments. When stimulated with insulin, 3T3-L1-shMCT1 cells showed significantly increased levels of glucose uptake, indicating that these cells maintain some insulin sensitivity; however, they did not respond to insulin as robustly as WT 3T3-L1 cells or show any increase in the insulin-stimulated glucose uptake following the 72 h treatment with 1 µM AZD3965 (Figure 5C). To ensure that the somewhat diminished insulin sensitivity of 3T3-L1-shMCT1 cells was a result of MCT1 expression inhibition and not a product of the lentiviral transduction system, 3T3-L1 cells containing shScramble were also characterized. Similar to WT cells, shScramble 3T3-L1 cells robustly enhanced the glucose uptake following insulin stimulation, and this effect was augmented by a 72 h treatment with 1 µM AZD3965 (Figure 5D).

Finally, to test the overarching hypothesis that MCT1 inhibition could induce hyperplasia in differentiated adipocytes, changes in the total triglyceride content of adipocytes cultured with or without insulin (100 nM) for 72 h following pretreatment for up to 72 h with or without AZD3965 (1 µM) were assessed. Following the treatment with AZD3965, cell culture media was replaced with media lacking AZD3965, with or without insulin. After an additional 72 h incubation, triglycerides where quantified. In support of our hypothesis, it was demonstrated that cells pretreated with AZD3965 accumulated significantly more triglycerides than adipocytes which had not been exposed to the MCT1 inhibitor when cultured without insulin (Figure 5G). As expected, all conditions (with or without AZD3965 pretreatment) showed a significant increase in total triglycerides when cultured with insulin, an agent known to promote lipogenesis. Moreover, adipocytes pretreated with AZD3965 for 48 h prior to the insulin stimulation developed significantly more triglycerides than AZD3965-naive cells also cultured with insulin (Figure 5G). However, this trend did not reach statistical significance for adipocytes pretreated with the MCT1 inhibitor for 24 h or 72 h. Together these results suggest that the AZD3965 treatment could induce hyperplasia and support an increase in the lipid storage capacity.

## 3. Discussion

Obesity is associated with adipose tissue expansion and ectopic fat deposition. Adipose tissue is a critical regulator of the whole-body metabolism and energy homeostasis, where its primary function is to store energy in the form of lipids with the glucose uptake playing a key role in providing substrates for lipogenesis [39]. In the context of a positive energy balance, where the nutrient intake exceeds energy expenditure, excess energetic substrates stored as triglyceride lipid droplets induce adipose tissue expansion. This expansion can occur by existing adipocytes increasing in size (hypertrophy) or by recruiting adipose progenitor cells for adipogenesis, leading to an increase in the adipocyte number (hyperplasia). Hence, adipocyte hypertrophy occurred to meet the demands for additional energy storage until the adipocyte reached a critical size theshold and capacity for lipid storage [40]. Subsequently, the adipocyte progenitor cell recruitment and expansion occurred by hyperplasia to accommodate the excess until the readily available adipocyte progenitor cells were depleted. These newly formed adipocytes could also expand until they reached their hypertrophic capacity at which time excess lipids would ‘spill over’ and were deposited at ectopic sites such as the liver, skeletal muscle, kidney, and pancreas [41].

It has been demonstrated that lean individuals predisposed to hypertrophic adipose tissue expansion have a reduced insulin sensitivity [42], and that adipocyte hypertrophy is a common feature seen in non-obese patients with T2DM [43]. In ‘metabolically fit’ individuals, adipose tissue expands primarily by hyperplasia. Adipose tissue expansion though hyperplasia is associated with an improved metabolic health, and, as such, therapeutically targeting hyperplasia has been suggested as a potential therapy for metabolic disease [6]. Moreover, it is well established that insulin sensitivity is directly correlative with the adipocyte size, wherein newly formed smaller adipocytes are insulin sensitive, and hypertrophic adipocytes are often insulin resistant [42,43].

Another important feature of adipocytes also associated with the glucose uptake is the production and release of lactate [44]. Lactate production, utilization, and signaling have extensive impacts on the metabolic health, particularly in adipose tissue depots [45]. Recently, it has been reported that via MCT1, the main lactate transporter in adipocytes, exogenous lactate administration can induce adipose tissue plasticity and adaptive thermogenesis [21,24]. It was demonstrated that these changes were the direct result of lactate-induced redox pressure as excess lactate fueled the production of pyruvate and NADH, at the expense of NAD^+^. As the glycolytic production of lactate and its transport via MCTs control the metabolic activity of fat cells, to gain further mechanistic insight into the influence of MCT1 activity on adipocyte biology, the classic adipocyte model cell system, differentiated 3T3-L1 cells, was employed. Using this model, a combination of in vitro and transcriptomic analyses of preadipocytes and mature adipocytes treated with and without the potent and selective MCT1 small-molecule inhibitor, AZD3965, was performed. Of note, AZD3965 is currently in clinical trials (NCT01791595) for the treatment of advanced cancers, including diffuse large B-cell lymphoma (DLBCL) and Burkitt lymphoma (BL). Phase I of this trial was completed in November 2020, and results regarding the safety and tolerability of AZD3965 are anticipated.

Inducing 3T3-L1 differentiation increased triglyceride synthesis, and ~4 days after the first exposure to differentiation medium, cells showed signs of lipid accumulation. However, when 3T3-L1 cells were differentiated in the presence of a pharmacological inhibitor of MCT1 (AZD3965), cells accumulated significantly less lipid than the untreated adipocytes (Figure 1D,E); the same observation was determined in the shMCT1-harboring cells (Figure 1A–C), suggesting that MCT1 inhibition impaired the lipid accumulation and differentiation. Exposure to differentiation media also induced transcriptional activation, and the up regulation of adipogenic genes such as the transcription factor and nuclear hormone, PPARγ. PPARγ positively regulates the expression of multiple genes involved in adipogenesis, including FABP4 and HSL [28]. FABP4 is a cytoplasmic carrier protein for fatty acids, and it has been proposed that FABP4 enables HSL activity in adipocytes by chaperoning lipids liberated by HSL-mediated lipolysis to the plasma membrane [46]. When the expression of these well-known markers of differentiation was assessed, a decrease in the expression levels of PPARγ, FABP4, and HSL was observed in AZD3965-treated and shMCT1-expressing cells, corroborating the previous finding reported herein that MCT1 inhibition impaired adipocyte differentiation (Figure 1F,G).

Mature (fully differentiated) adipocytes treated with AZD3965 also exhibited a reduced lipid content over the course of the AZD3965 treatment (Figure 2A,B). It was posited that this decrease in the lipid droplet content could have been the result of a decrease in lipogenesis, an increase in lipolysis, or adipocyte de-differentiation, a process that has been described by others [47,48], involving a rapid ‘liposecretion’ event [49]. To gain insight into the mechanisms that are governed by MCT1 inhibition, changes in NADP^+^(H) bioavailability (a critical factor for lipogenesis), the intracellular glycerol content (the major product of lipolysis), adipokine secretion, as well as transcript levels and protein expression of the previously mentioned adipogenic proteins were assessed. MCT1 inhibition significantly reduced intracellular concentrations of both reduced and oxidized forms of NADP^+^(H), yet this decrease was marginal with a less than 15% decrease observed (Figure 2D), suggesting that other mechanisms could contribute to the significant decrease in lipid observed, following MCT1 inhibition in adipocytes (Figure 2A,B). Indeed, when AZD3965-treated adipocytes were probed for changes in lipolysis, MCT1 inhibition led to a large increase in intracellular glycerol and, thus, an increase in cellular lipolysis (Figure 2E). Although this increase in lipolysis could account for the decrease in the adipocyte lipid content, it was critical to rule out the possibility of adipocyte de-differentiation by assessing changes in transcript levels, the expression of adipogenic proteins, and adipokine secretion.

The RT-qPCR analysis revealed that the mRNA levels for PPARγ and HSL were both significantly decreased following 24 h of the AZD3965 treatment (Figure 2F). This was reflected at the protein level by a decrease in the HSL protein expression following 72 h of MCT1 inhibitor treatment; however, no changes in PPARγ expression were observed (Figure 2G). Conversely, FABP4 mRNA and protein expression were significantly increased following 24 h and 48 h of the AZD3965 treatment, respectively (Figure 2F,G). Despite the decreased HSL expression, the maintained PPARγ and elevated FABP4 expression suggest that adipocytes remained differentiated following MCT1 inhibition. To further establish that adipocytes remained differentiated following the AZD3965 treatment, conditioned media collected from adipocytes pre-treated with or without AZD3965 for 72 h were subjected to an adipokine antibody array. No significant changes in adipokine secretion following MCT1 inhibition were detected (Figure 2H), supporting the notion that de-differentiation did not occur. Taken together, these results suggest that an increase in lipolysis following the AZD3965 treatment accounted, at least in part, for the observed decrease in the lipid content. However, further experiments would be required to fully elucidate the influence that MCT1 inhibition has on the lipid metabolism, including lipogenesis and fatty acid β-oxidation.

The acute physiological consequence of MCT1 inhibition is an intracellular lactate accumulation owing to the cells’ inability to export lactate via its primary transporter. Therefore, changes in intracellular lactate in adipocytes following incubation with AZD3965 at discrete time points ranging from 15 min to 24 h were monitored. Indeed, treatment with the MCT1 inhibitor rapidly increased the intracellular lactate; however, after prolonged exposure to AZD3965, this effect was greatly diminished (Figure 3A). To explore the potential fates of lactate which can be metabolized though its oxidation into pyruvate or complete oxidation by the mitochondria, intracellular pyruvate and the NAD^+^/NADH ratio were also monitored over the same time course. An acute, significant increase in intracellular pyruvate was observed (Figure 3B) as was a significant decrease in the relative NAD^+^/NADH ratio (Figure 3C). This decrease in the NAD^+^/NADH ratio indicated that there was an increase in the intracellular lactate relative to pyruvate, and that MCT1 inhibition induced reductive stress. This observation was in agreement with the finding that elevated levels of intracellular lactate also induce redox stress in adipocytes as reported by others [21]. Taken together, these results suggested that although there was an increase in both the lactate and pyruvate, the increase in lactate was more robust; therefore, it is unlikely that the excess lactate was oxidized back into pyruvate, but rather, may have been oxidized directly by the mitochondria.

To gain insight into the transcriptional and molecular processes influenced by MCT1 inhibition, a transcriptomic analysis (RNA-seq) was performed on mature adipocytes treated with or without AZD3965 for 24 h. In total, 277 differentially expressed genes (DEGs) were identified though a comparison of the gene expression profiles from the treated versus untreated adipocytes. A molecular functions analysis in IPA revealed a transcriptional signature characterized by the upregulation of mRNAs related to the progression of cell cycle (Figure 3D,E). Previously, it has been suggested that mature adipocytes may retain the ability to re-enter the cell cycle and proliferate. Evidence of this phenomenon comes from Xu et al., who recently captured adipocyte proliferation on a phase contrast microscope [50]. While the low resolution of these videos leaves unanswered questions as to the mechanism of proliferation (i.e., de-differentiation or direct division of adipocytes), it is clear that cells with lipid droplets do retain the capacity to divide. To explore the possibility that MCT1 inhibition could promote re-entry of the mature adipocytes into the cell cycle as suggested by our transcriptomic analysis, mRNA levels of well characterized, cell cycle-promoting genes were assessed, revealing a significant increase in transcript levels of proliferation markers, CDK1 (cyclin-dependent kinase 1), PLK1 (polo-like kinase 1), TOP2α (DNA topoisomerase alpha), and cyclin B, following AZD3965 treatment (Figure 4A). Furthermore, the active form of CDK1, a central regulator that drives cells though the cell cycle [29], was found to be increased following MCT1 inhibition (Figure 4B,C).

When probed directly, it was shown that MCT1 inhibition promoted adipocyte proliferation specifically in differentiated 3T3-L1 cells, but not in preadipocytes or 3T3-L1-shMCT1 cells (Figure 4D,E). To visualize this proliferation, an immunocytochemistry experiment probing the expression of the proliferation marker, Ki67, following 24 h treated with AZD3965 was performed and quantified. Ki67 expression was observed in cells containing lipid droplets and was significantly increased in MCT1 inhibitor-treated cells (Figure 4F–H). These findings strongly support the premise that mature, lipid-laden adipocytes have the potential to re-enter the cell cycle following MCT1 inhibition. However, without monitoring mitosis though successful cytokinesis, we cannot rule out the possibility that our results could represent endoreplication resulting in polyploidy, rather than proliferation. Indeed, it has been demonstrated that differentiated 3T3-L1 adipocytes can become multi-nucleated [50].

As proliferation in mature adipocytes is a recently recognized mechanism of insulin sensitizing that accompanies hyperplasia, the insulin-stimulated glucose uptake was assessed following the MCT1 inhibitor treatment. Following up to 72 h of the inhibitor treatment, no significant changes in the basal, non-insulin-stimulated glucose uptake were observed in WT 3T3-L1 cells (Figure 5A) or shMCT1 containing 3T3-L1 cells (Figure 5D). However, unlike shMCT1, 3T3-L1 showed a significant increase in the insulin-stimulated glucose uptake following ≥48 h of AZD3965 (1 µM) treatment (Figure 5B,E). Moreover, it was shown that this effect was CDK1-dependent as it could be blocked with the addition of RO-3306, a small molecule CDK1 inhibitor (Figure 5C). The evaluation of 3T3-L1-shMCT1 cells revealed that although these adipocytes were insulin responsive, the insulin-stimulated glucose uptake was attenuated in these cells. As shScramble-expressing 3T3-L1 cells showed insulin responsiveness similar to that of WT cells (Figure 5F), it appeared that the MCT1 knockdown may have interfered with the development of insulin-sensitive, mature adipocytes.

The ability of MCT1 inhibition to enhance the adipocyte lipid storage capacity (the physiological function of hyperplasia) was assessed. To probe changes in the adipocyte lipid storage capacity, intracellular triglycerides were quantified in adipocytes following pretreatment with or without AZD3965 for up to 72 h. Indeed, adipocytes exposed to the MCT1 inhibitor for 24 h, 48 h, and 72 h all contained significantly more triglycerides than adipocytes, which had not received the AZD3965 pretreatment (Figure 5G). Moreover, when cultured for 72 h with 100 nM insulin, a potent activator of lipogenesis, adipocytes pretreated with the MCT1 inhibitor for 48 h accumulated significantly more triglycerides compared to adipocytes which had not been exposed to AZD3965 (Figure 5G). Adipocytes pretreated for 24 h and 72 h also trended toward an increase in the triglyceride content; however, this increase did not reach statistical significance. As these adipocytes were incubated with insulin, it is also possible that they may have reached their hypertrophic capacity, limiting the ability of adipocytes to accumulate additional triglycerides.

Collectively, our data showed that MCT1 inhibition has profound effects on the adipocyte metabolism. Specifically, treatment with the MCT1 inhibitor AZD3965 enhanced lipolysis, promoted adipocyte proliferation, augmented the insulin-stimulated glucose uptake, and enhanced the triglyceride storage capacity, providing compelling evidence that MCT1 inhibition may promote adipocyte hyperplasia. However, to fully elucidate the influence that MCT1 inhibition has on the systemic metabolism and the potential therapeutic benefit of AZD3965 in the context of metabolic disease, additional in vitro and in vivo experiments characterizing changes in adipose tissue morphology and metabolism and systemic metabolism are required. As previously mentioned, AZD3965 is currently in clinical trials (NCT01791595) for advanced cancers. This trial represents the first time in patients for this class of drug to define the maximum tolerated dose and proof of mechanism. However, monitoring the impacts of treatment on the adipocyte morphology, insulin sensitivity, and systemic metabolism in trial participants could be particularly informative. The expansion of adipose tissue mass by excessive hypertrophy and ectopic lipid accumulation is a major contributor to metabolic disease in obesity. By contrast, promoting the expansion of adipose tissue though hyperplasia has the potential to redistribute excess lipid between newly differentiated adipocytes with enhanced insulin sensitivity and lipid storage capacity. Thus, improving adipocyte function via MCT1 inhibition-induced hyperplasia may provide a novel therapeutic strategy for obesity and related metabolic disorders.

## 4. Materials and Methods

### 4.1. Cell Culture

The 3T3-L1 cells (CRL-173™; ATCC^®^, Manassas, VA, USA) were cultured in ‘complete media’ containing Dulbecco’s DMEM with 10% iron fortified, calf serum (30-2030™; ATCC^®^, Manassas, VA, USA), and 1% Pen-Strep. All cells were maintained at 37 °C with 5% CO_2_ atmosphere in a humidified incubator. Media was renewed every 2 days and cells were passaged by splitting at a 1:10 ratio to ensure confluency did not exceed 80%. Cells were maintained below passage 8.

### 4.2. Cell Line Development

Lenti plasmid vectors containing shNA targeting MCT1 or a shScramble sequence were purchased from GeneCopoeia (Rockville, MD, USA). Lentiviral particles were produced using HEK293T cells and a third-generation packaging system, MISSION Lentiviral Packaging Mix, as per manufacturer’s (Sigma-Aldrich, Saint Louis, MO, USA) recommendations. To generate shMCT1 cells, 3T3-L1 cells were seeded and transduced with optimized titers of freshly harvested lentivirus. Twelve hours after transduction, medium was changed and cells were allowed to recover for 24 h before being placed under antibiotic selection (2 µg/mL puromycin) for 3–6 days. Cells surviving antibiotic selection were harvested for both reverse transcription–quantitative chain polymerase reaction (RT-qPCR) and immunoblotting analyses.

### 4.3. Differentiation Protocol

The 3T3-L1 differentiation protocol was adapted from Green and Meuth (1974) [25,51]. Cells were seeded at ~80% confluency and allowed to reach confluency prior to induction of differentiation. Then, 1–2 days post confluency, growth media was changed to ‘day 0′ media consisting of media supplemented with 0.5 mM 1-methyl-3-isobutyl xanthine (IBMX), 1 µM dexamethasone, and 10 µg/mL insulin. On ‘day 3’, media was changed to growth media with 10 µg/mL insulin only, and on ‘day 6’, media was replaced with normal growth media. After day 6, the complete media was refreshed every 2 days. Using Oil Red O staining, RT-qPCR, and immunoblotting, it was determined that 3T3-L1 cells were fully differentiated into lipid-laden adipocytes by ‘day 8’ of the protocol, as reported by others [18,52]. All differentiation experiments were performed on cells between passages 4 and 6 to ensure differentiation efficiency was consistent.

### 4.4. Oil Red O Staining

Cells were then fixed with 4% PFA for 10 min. After fixation, PFA was removed; cells washed 3× with PBS and allowed to dry completely. The lipid-specific stain Oil Red-O was dissolved in isopropanol to a concentration of 12 mM, was filtered with a 0.2-micron filter, and further diluted 6:4 with distilled water. Following 20 min of incubation, the stain solution was filtered again (0.2-micron filter) and added to fixed cells for 10 min. Subsequently, stain was then removed and cells were washed thoroughly with distilled water. Consequently, 10x images of cells were taken from randomized fields of view with the Cytation 5 Cell Imaging Multi-Mode reader (BioTek Instruments, Winooski, VT, USA). Lipid content was also quantified on the Cytation 5.

### 4.5. RT-qPCR

RNA was harvested from preadipocytes using the RNeasy^®^ Plus Mini Kit as per manufacturer’s recommendations (QIAGEN, Hilden, Germany). For differentiated adipocytes, RNA was collected and isolated using TRIzol (Thermo Fisher Scientific, Waltham, MA, USA) according to the manufacturer’s instructions. Quantification of RNA was performed using the Nanodrop 1000 Spectrophotometer. RNA (2 µg) was reverse transcribed using Superscript III First Strand synthesis kit (Invitrogen, Waltham, MA, USA). RT-qPCR detection with SYBR green was performed with the resulting cDNA and a 50/50 forward primer: reverse primer ratio using the QuantStudio™ 5 (Applied Biosystems™, Waltham, MA, USA). Oligonucleotide primer sequences used in this study can be found below.

### 4.6. Immunoblotting

For the collection of membrane-bound proteins (i.e., MCT1), cells were lysed and the membrane fraction of protein was isolated as previously described [53]. Briefly, cells were lysed with ice cold HES buffer (250 mM sucrose, 20 mM HEPES, 1 mM EDTA, pH7.4) containing a cocktail of protease and phosphatase inhibitors (Roche Diagnostic, Basel, Switzerland) and centrifuged for 5 min (500 g at 4 °C) to pellet out nuclei. The remaining supernatant (containing cytosolic and membrane-bound proteins) was collected. For all other immunoblotting experiments, RIPA lysis buffer containing protease and phosphatase inhibitors was used, and protein was isolated via centrifugation at 17,000× *g* for 10 min at 4 °C. For all samples, protein concentration of the lysates was measured using the BCA Protein Assay Kit (Pierce Biotechnologies, Waltham, MA, USA). Diluted lysates were added to Laemmli sample buffer and denatured at 95 °C for 5 min. After sample preparation, proteins were resolved via SDS-polyacrylamide gel electrophoresis (4–12%) using NuPAGE 4–12% Bis-Tris gels (Invitrogen, Waltham, MA, USA) and transferred to nitrocellulose membranes. Blots were placed in blocking buffer (LI-COR Biosciences, Lincoln, NE, USA) and incubated overnight at 4 °C with primary antibodies at the recommended dilution. Blots were washed 3 times (5 min per wash) in TBST (20 mM Tris, pH 7.6, 140 mM NaCl, and 0.1% TWEEN-20), and then incubated with the appropriate IRDye-conjugated secondary antibody (LI-COR Biosciences, Lincoln, NE, USA) diluted in blocking buffer and incubated at room temperature for 1 h. After 3 washes (5 min per wash) with TBST, blots were revealed using the LI-COR Odyssey CLx system. Bands were quantified using Image Studio™ (LI-COR Biosciences Lincoln, NE, USA).

### 4.7. Cell Viability

The 3T3-L1 cells were seeded and differentiated in white, clear-bottom, 384-well plates (Corning^®^, Corning, NY, USA). Following differentiation, adipocytes were treated for 24, 48, or 72 h with varying concentrations of AZD3965 ranging from 0.1 nM to 10 µM. Following the indicated incubation period, cells were assayed with CytoTox-Glo™ (Promega^®^, Madison, WI, USA) according to the manufacturer’s instructions to assess cell toxicity. Briefly, this assay is a luminescent cytotoxicity kit that measures the relative number of dead cells in a population though the addition of a cell impermeant luminogenic substrate which luminesces in the presence of ‘dead-cell protease’, a protein released from membrane-compromised dead cells. Luminescence was read on a spectrophotometer (FlexStation 3; Molecular Devices, San Jose, CA, USA). The data generated were normalized by dividing the relative luminescence units (RLU) collected for each experimental condition by the average RLU of ‘basal’ untreated cells considered to be 100% viable.

### 4.8. NADP^+^ + NADPH Bioavailability

The 3T3-L1 cells were seeded, differentiated, and treated with or without AZD3965 (1 µM) for up to 72 h in clear 96-well plates (Corning^®^, Corning, NY, USA). Following treatment, the NADP^+^/NADPH-Glo™ (Promega^®^, Madison, WI, USA) kit was used to measure the total NADP^+^ + NADPH concentration within each sample according to the manufacturer’s instructions. In the presence of either NADP^+^ or NADPH, the enzyme ‘reductase’ reduced a proluciferin reductase substrate to form luciferin, which emitted a luminescent signal directly proportional to the quantity of NADP^+^ and NADPH. Luminescence was read on a spectrophotometer (FlexStation 3; Molecular Devices, San Jose, CA, USA).

### 4.9. Lipolysis (Intracellular Glycerol)

The 3T3-L1 cells were seeded, differentiated, and treated with or without AZD3965 (1 µM) for up to 72 h in clear 96-well plates (Corning^®^, Corning, NY, USA). After media removal and washing cells with PBS, the Glycerol-Glo™ (Promega^®^, Madison, WI, USA) assay kit was used to assess changes in lipolysis though the quantification of intracellular glycerol according to the manufacturer’s instructions. A luminescent signal was produced that was proportional to the amount of glycerol within the sample. Luminescence was read on a spectrophotometer (FlexStation 3; Molecular Devices, San Jose, CA, USA).

### 4.10. Adipokine Antibody Array

The 3T3-L1 cells were differentiated and treated with or without AZD3965 for 72 h. After this incubation, media was replenished, and adipocytes were cultured for an additional 24 h to allow for adipokine secretion and media conditioning. Media was collected and analyzed for adipokine secretion with the Proteome Profiler Mouse Adipokine Array Kit (R&D Systems, Upper Midwest, MN, USA) according to the manufacturer’s instructions. Chemiluminescence was detected with the LI-COR Odyssey^®^ XF Imaging System with a 2 min exposure time. Positive signals were quantified using Image Studio™ (LI-COR Biosciences, Lincoln, NE, USA) to quantify signal intensity. Data presented represent signal intensity normalized by the signal intensity of reference antibodies within the array.

### 4.11. Intracellular Lactate

Intracellular lactate was measured using the Lactate-Glo™ Assay Kit (Promega^®^ Madison, WI, USA) according to the manufacturer’s instructions. The 3T3-L1 cells were seeded and differentiated in white, clear-bottom, 96-well plates (Corning^®^, Corning, NY, USA). After differentiation, cells were treated with compound or vehicle as indicated. Following 15 min, 30 min, 1 h, 4 h, 8 h, or 24 h incubation with 1 µM AZD6965, media was removed and cells were lysed. Lysates were then subjected to a proprietary enzymatic reaction coupling lactate oxidation and NADH production with a bioluminescent detection system supplied by the Lactate-Glo™ Assay kit, and luminescence was read on a spectrophotometer (FlexStation 3; Molecular Devices, San Jose, CA, USA). The data generated were normalized by dividing the relative luminescence units (RLU) collected for each experimental condition by the average RLU of vehicle control.

### 4.12. Intracellular Pyruvate

Intracellular pyruvate was measured using the Amplite™ Fluorimetric Pyruvate Assay Kit (AAT Bioquest, Sunnyvale, CA, USA) according to the manufacturer’s instructions. The 3T3-L1 cells were seeded and differentiated in white, clear-bottom, 96-well plates (Corning^®^, Corning, NY, USA). After differentiation, cells were treated with compound or vehicle as indicated. Following 15 min, 30 min, 1 h, 4 h, 8 h, or 24 h incubation with 1 µM AZD6965, media was removed and cells were lysed with ReadiUse™ mammalian cell lysis buffer (AAT Bioquest, Sunnyvale, CA, USA). Lysates were then subjected to an enzyme-coupled reaction with the Quest Fluor™ Pyruvate Sensor, resulting in an absorbance signal detected at 575 nm which was proportional to the quantity of pyruvate within the sample. Absorbance was read on a spectrophotometer (FlexStation 3; Molecular Devices, San Jose, CA, USA).

### 4.13. NAD^+^/NADH Ratio

The 3T3-L1 cells were seeded, differentiated, and treated with or without AZD3965 (1 µM) for 15 min, 30 min, 1 h, 4 h, 8 h, or 24 h in clear 96-well plates (Corning^®^, Corning, NY, USA). Following incubation with the compound, the NAD^+^/NADH-Glo™ (Promega^®^, Madison, WI, USA) kit was used to measure both the NAD^+^ and NADH concentrations within each sample according to the manufacturer’s protocol. Briefly, samples were lysed and split into 2 separate, white, 96-well plates (Corning^®^, Corning, NY, USA). One plate was treated with a strong acid (0.4 N HCl) to eliminate NADH in the sample, while the other plate was treated with a strong base (0.2 N NaOH) to eliminate NAD^+^, and both plates were incubated at 60 °C for 15 min. Samples were then neutralized and subjected to the NAD^+^/NADH-Glo™ assay. Within this assay, in the presence of NAD^+^ or NADH, the enzyme ‘reductase’ reduced a proluciferin reductase substrate to form luciferin, which emitted a luminescent signal directly proportional to the quantity of NAD^+^ or NADH within the sample. Luminescence was read on a spectrophotometer (FlexStation 3; Molecular Devices, San Jose, CA, USA). To calculate the NAD^+^/NADH ratio for each sample, luminescence from acid-treated samples (reflecting NAD^+^ level) was divided by the luminescence signal from the corresponding base-treated samples (reflecting NADH levels).

### 4.14. RNA-Seq

3T3-L1 cells were seeded in 10 cm^2^ dishes at 70% confluency and subjected to differentiation for 8 days. Differentiated cells were treated with vehicle (DMSO) or MCT1 inhibitor (1 µM AZD6965) for 24 h. Following treatment, cells were collected, and RNA was isolated using TRIzol (Thermo Fisher Scientific, Waltham, MA, USA) according to the manufacturer’s protocol. Subsequently, RNA samples were purified using the RNeasy^®^ Plus Mini Kit RNA clean up protocol as per manufacturer’s (QIAGEN) recommendations. Quantification of RNA was performed using the Nanodrop 1000 Spectrophotometer. All RNA samples were shipped to Novogene (Sacramento, CA, USA) for mRNA sequencing (RNA-seq) using the Illumina NovaSeq platform with paired-end 150 bp (PE 150) sequencing and data quality control strategy. The raw reads were then aligned and mapped to the build mm10 mouse reference genome using STAR (spliced transcripts alignment to a reference) software. The total mapping rate was over 80% for all samples in the dataset. Gene expression level was quantified by calculating FPKM (fragments per kilobase of transcript sequence per millions base pairs sequenced) from the STAR mapping files. A differential expression analysis was performed using the R package DESeq2. *p* values generated by multiple binomial tests were adjusted with the Benjamini and Hochberg approach for controlling false discovery and reported as the adjusted *p* value (padj). The theshold for statistical significance was defined as padj < 0.05.

### 4.15. Ingenuity^®^ Pathway Analysis (IPA)

For pathway analysis, changes in transcript FPKM and significance values resulting from the RNA-seq experiment were uploaded and processed using QIAGEN Ingenuity^®^ Pathway Analysis software (QIAGEN IPA). After each annotated gene was mapped to its corresponding gene object in the IPA Knowledge Base, molecular functions analysis was performed to identify significantly (padj < 0.05) altered biological functions.

### 4.16. Confocal Microscopy

Cells were seeded and differentiated on 8-chamber µ-Slides (Ibidi^®^, Gräfelfing, Germany). Differentiated cells treated with vehicle (DMSO) or 1 µM AZD3965 for 24 h were fixed in 4% PFA for 10 min at room temperature, permeabilized with 0.1% Triton™ X-100 in PBS, and blocked with 3% BSA in PBS for 1 h. Cells were, subsequently, incubated with Ki67 antibody (NBP2-22112; Novus Biologicals, Littleton, CO, USA) at a 1:500 dilution in PBS 3% BSA overnight at 4 °C. Cells were washed with PBS and incubated with goat anti-rabbit Alexa Fluor 647 secondary antibody (Invitrogen; Waltham, MA, USA) at 1:2000 dilution in PBS 3% BSA for 1 h. Lipid droplets were stained with BODIPY^®^ (Invitrogen, Waltham, MA, USA) for 20 min at room temperature according to the manufacturer’s recommendations. Nuclei were stained for 20 min at room temperature with 4,6-diamidino-2-phenylindole (DAPI) (Sigma-Aldrich, St. Louis, MO, USA) in PBS 3% BSA, diluted at 1:5000 from a 1 mg/mL stock. Images were acquired on the Leica TCS SP8 laser scanning confocal microscope (Leica Microsystems, Wetzlar, Germany) at a magnification of 630× with 3× zoom.

### 4.17. Ki67 Expression Quantification

Slides were prepared as outlined in ‘Confocal Microscopy’ and imaged and analyzed on the Cytation 5 Cell Imaging Multi-Mode reader (BioTek Instruments, Winooski, VT, USA). After staining, Ki67 expression was imaged and analyzed on the Cytation 5 Cell Imaging Multi-Mode reader though the measure of fluorescence intensity and deep red fluorescent (Alexa647) positive pixels. This value was then normalized by the total number of nuclei as indicated by DAPI staining and the ‘cell counting’ image analysis program on the Cytation 5, resulting in the average Ki67 expression per cell for each 1000 nuclei captured in 12 randomized images per condition at 10× magnification.

### 4.18. Proliferation

The 3T3-L1 cells were seeded and differentiated in black, clear-bottom, 96-well plates (Corning^®^, Corning, NY, USA). After differentiation, wells were treated with vehicle or AZD3965 concentrations ranging from 10 nM to 10 µM for 24 h, 48 h, or 72 h, as indicated. Cell viability was measured at the end of each treatment time using the CyQUANT™ NF Cell Proliferation assay (Invitrogen™, Waltham, MA, USA) according to the manufacturer’s protocols.

### 4.19. Glucose Uptake

The 3T3-L1 cells were seeded and differentiated in clear-bottom 96-well plates (Corning^®^, Corning, NY, USA). After differentiation, cells were treated with or without 1 µM AZD3965 for 24 h, 48 h, or 72 h, as illustrated in Supplementary Figure S2 (schematic outline of experimental procedure). Cells were incubated in serum-free media the night before performing the assay. The following morning, media was changed to serum and glucose-free media for 2 h prior to the experiment. Cells were then stimulated with 175 nM insulin or vehicle (PBS) for 30 min. Glucose uptake was measured using the Glucose Uptake-Glo™ Assay (Promega^®^) according to the manufacturer’s protocol. The data generated were normalized by dividing the relative luminescence units (RLU) measured in insulin-stimulated wells by vehicle-treated controls. The aforementioned experiment was repeated in the presence of the CDK1 inhibitor, RO-3306 (10 µM), where indicated (Figure 5C).

### 4.20. Hyperplasia (Intracellular Triglycerides)

The 3T3-L1 cells were seeded and differentiated in clear-bottom 96-well plates (Corning^®^, Corning, NY, USA). After differentiation, cells were treated with or without 1 µM AZD3965 for 24 h, 48 h, or 72 h. After compound incubation, media was changed to normal growth media with or without 100 nM insulin and cultured for an additional 72 h. Upon completion of the second incubation period, adipocytes were lysed and subjected to the Triglyceride-Glo™ Assay (Promega^®^, Madison, WI, USA) according to the manufacturer’s instructions. The luminescent signal produced was proportional to triglycerides detected within the sample. Luminescence was read on a spectrophotometer (FlexStation 3; Molecular Devices, San Jose, CA, USA).

### 4.21. Statistics

Data analysis was performed using Prism 8 software (GraphPad Software Inc., San Diego, CA, USA). All statistical significance was generated based on Student’s *t*-test or one way ANOVA as appropriate for multiple comparisons and a significance value of at least *p* < 0.05. Unless otherwise stated, all data represent the average of 3 experimental replicates ± the standard error of the mean (SEM).

## Data Availability

Data available on request. The data presented in this study are available on request from the corresponding author.

## Supplementary Materials

The following are available online at https://www.mdpi.com/article/10.3390/ijms23031901/s1.
