# Supplementary material for: Inhibition of the Monocarboxylate Transporter 1 (MCT1) Promotes 3T3-L1 Adipocyte Proliferation and Enhances Insulin Sensitivity
Source: Int J Mol Sci. 2022 Feb 8;23(3):1901. doi: 10.3390/ijms23031901 (PMC8836706; doi:10.3390/ijms23031901)
Supplement: Supplementary file 1 [file ijms-23-01901-s001.zip › ijms-1165144-supplementary.pdf]

Supplementary Figure S1

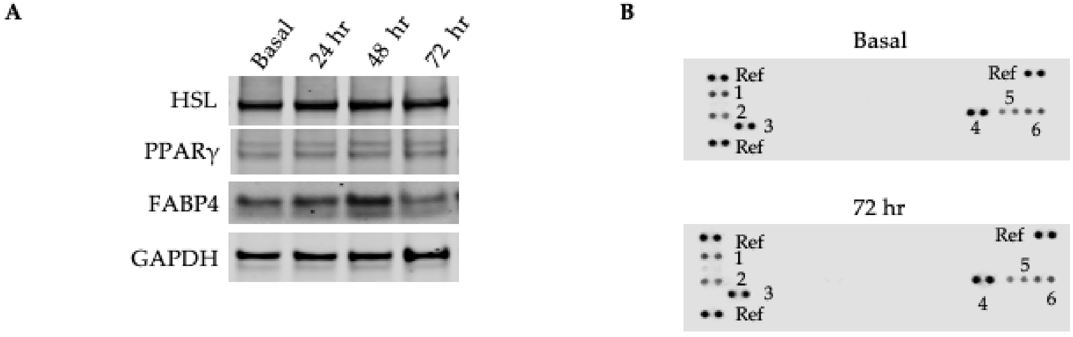

1. Adiponectin
2. MCP1
3. VEGF
4. Resistin
5. Serpin E1
6. TIMP1

Supplementary Figure S1. Protein and Metabolite Analysis of 3T3-L1 Cells. (A) Cell lysates of adipocytes treated with or without 1μM AZD3965 for 24 hr, 48 hr and 72 hr were subjected to immunoblotting experiments with antibodies for the detection of HSL, PPARγ, FABP4 and GAPDH. Results were quantified and presented in Figure 2G. (B) Differentiated adipocytes were treated with or without 1 μM AZD3965 for 72 hr as indicated. Fresh media was added and conditioned for 24 hr prior to its collection and analysis with an adipokine antibody array. Results were quantified and presented in Figure 2H.

Supplementary Figure S2

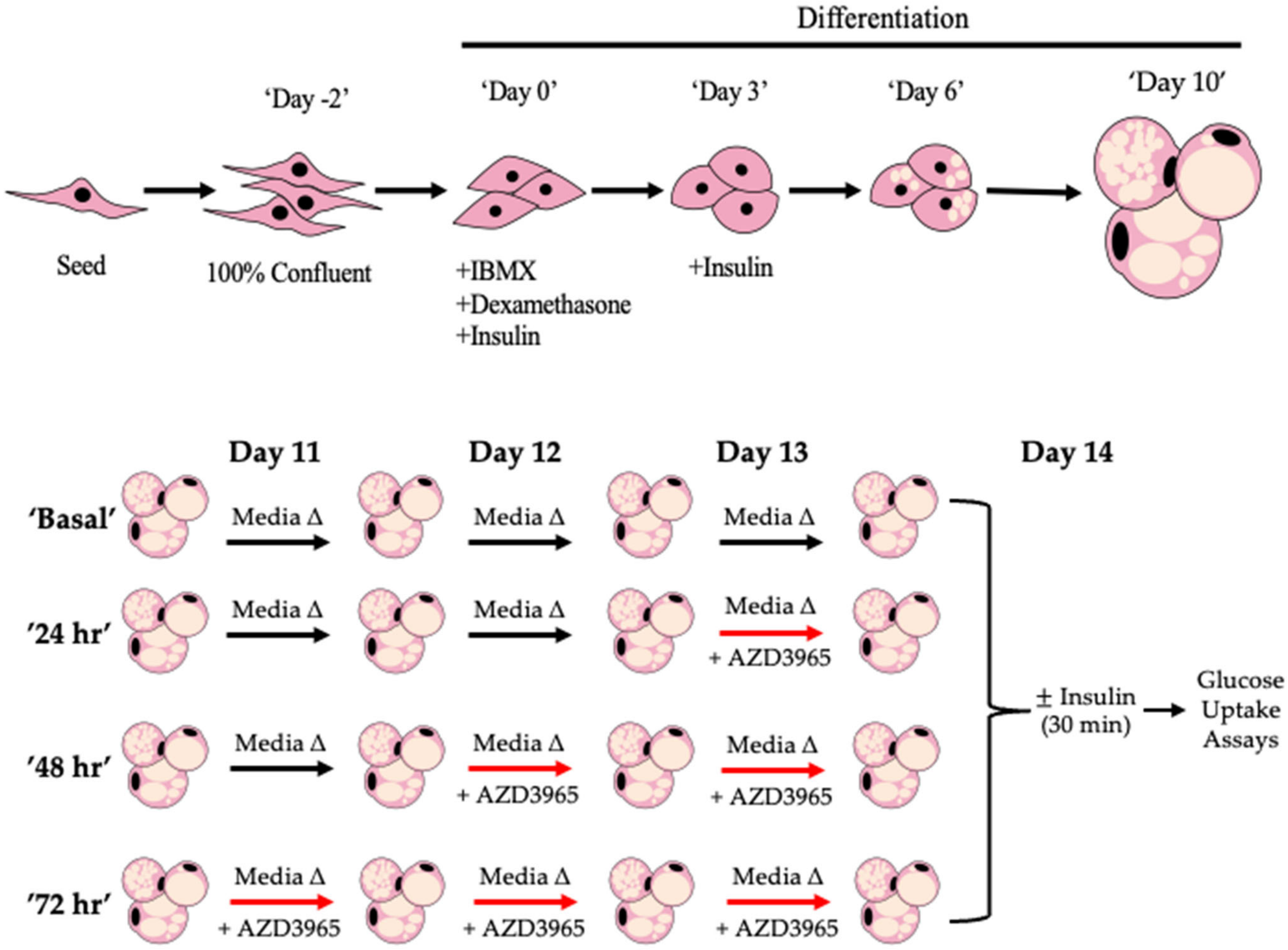

Supplementary Figure S2. Schematic Outline of Experimental Procedure. 3T3-L1 cells were grown to confluence and differentiated according to the time course above. Following differentiation, adipocytes were treated with or without AZD3965 for the time indicated such that all experiments were terminated and subsequent analysis performed on the adipocytes on the same 'day'.
